# Exploring the National Nursing Research Priorities in the Eastern Mediterranean Region and Overcoming the Associated Challenges: An Expert Opinion

**DOI:** 10.7759/cureus.64540

**Published:** 2024-07-14

**Authors:** Abdulqadir J Nashwan, Ebtsam A Abou Hashish, Ahmed S Mohamed, Intima Alrimawi, Ibrahim Aqtam, Salwa Al Obeisat, Fadwa Alhalaiqa, Mohammad Alzaatreh, Majdi Al Hadidi, Sadeq AL-Fayyadh, Jadeel N Faleh, Marwa Shaban, Mostafa Shaban, Alireza Mirzaei, Reza Vakilabad, Jalal Arabloo, Sulman Siddique, Aisha Shdefat, Maha Atout, Hanan F Alharbi

**Affiliations:** 1 Department of Nursing &amp; Midwifery Research, Hamad Medical Corporation, Doha, QAT; 2 Faculty of Nursing, Alexandria University, Alexandria, EGY; 3 College of Nursing, King Saud Bin Abdulaziz University for Health Sciences, Jeddah, SAU; 4 Department of Nursing, Hamad Medical Corporation, Doha, QAT; 5 School of Nursing, Georgetown University, Washington, DC, USA; 6 Ibn Sina College for Health Professions, Nablus University for Vocational and Technical Education, Nablus, PSE; 7 Faculty of Nursing, Jordan University of Science and Technology, Irbid, JOR; 8 College of Nursing, Qatar University, Doha, QAT; 9 Department of Applied Medical Sciences, Al-Balqa Applied University, Amman, JOR; 10 Department of Nursing, Al-Zaytoonah University, Amman, JOR; 11 Department of Adult Nursing, University of Baghdad, Baghdad, IRQ; 12 Al-Muthanah Health Directorate, Al-Muthanah Governorate, Al-Muthanah, IRQ; 13 Department of Community Health Nursing, Cairo University, Cairo, EGY; 14 Department of Gerontological Nursing, Faculty of Nursing, Cairo University, Cairo, EGY; 15 School of Nursing and Midwifery, Ardabil University of Medical Sciences, Ardabil, IRN; 16 School of Nursing and Midwifery, Guilan University of Medical Sciences, Rasht, IRN; 17 Health Management and Economics Research Center, Iran University of Medical Sciences, Tehran, IRN; 18 Department of Healthcare Education, Shifa International Hospitals, Islamabad, PAK; 19 Department of Nursing, Sultan Qaboos University (SQU), Muscat, OMN; 20 Faculty of Nursing, Philadelphia University, Amman, JOR; 21 Department of Nursing, Princess Nourah Bint Abdulrahman University, Riyadh, SAU

**Keywords:** research priorities, nursing practice, nursing research, eastern mediterranean region, national nursing research priorities

## Abstract

Background: Nurses play a significant role in contributing to various health priorities globally, including research. Identifying the status of national nursing research priorities in the Eastern Mediterranean Region is crucial to cultivating these priorities. This expert opinion paper highlights the existing status of national nursing research priorities in Eastern Mediterranean Region countries concerning their existence and publicity.

Methods: Experts from nine Eastern Mediterranean Region countries, including Egypt, Iran, Iraq, Jordan, Pakistan, Palestine, Qatar, Oman, and Saudi Arabia, contributed to this report. They participated by completing a cross-sectional survey and providing a narrative description of their opinions.

Results: The findings revealed that 58% of the participating countries have existing national nursing research priorities, while 25.8% do not, and 16% are under development. Governmental organizations developed the largest portion of the priorities (38%). Midwives were not considered in half of the published priorities. The vast majority of national nursing research priorities (65%) were developed by experts' opinions and consensus, and 33% only have an associated strategy, outcome measures, and/or funding opportunities. Generally, most published research priorities were not updated regularly.

Conclusion: Eastern Mediterranean Region countries face a challenge with the need for more nurses, which may hinder their involvement in research projects or continued education. Despite this, all countries involved in this report emphasized the importance of developing nursing education and research as priorities for improving their current nursing workforce. Health policymakers, nurse practitioners, academic researchers, educators, and nursing leaders should collaborate to develop operational plans to foster national nursing education and research.

## Introduction

The Eastern Mediterranean Region (EMR) is a geographical region encompassing parts of the Middle East, North Africa, and the Arabian Peninsula (comprising 22 member territories with a population of nearly 679 million) [1,2]. It includes countries such as Afghanistan, Bahrain, Djibouti, Egypt, the Islamic Republic of Iran, Iraq, Jordan, Kuwait, Lebanon, Libya, Morocco, Oman, Pakistan, Palestine, Qatar, Saudi Arabia, Somalia, Sudan, the Syrian Arab Republic, Tunisia, the United Arab Emirates, and Yemen. The World Health Organization (WHO) recognizes the EMR as one of its six regions and works to support health and healthcare systems in the region through programs, initiatives, and partnerships. The EMR is characterized by unique cultural, economic, and political challenges affecting the region's health and healthcare; the WHO works to address these challenges through evidence-based policies and programs [3]. There is no clear evidence of clinical nurses' and midwives' participation in creating a regional research agenda for the EMR [4].

Nurses have made significant contributions to a variety of health priorities around the world [5]. One of these priorities is research. Priorities in nursing research can help to inform practice, the profession, education, and policy [6]. Therefore, this must be involved in the national health agenda. The national health agenda can only be achieved if consistent and continuous efforts are made to maximize the contribution of nurses in their roles as members of interprofessional health teams. These efforts necessitate policy interventions that empower nurses, optimize their scope of practice, and promote their positive influences and effects on the quality of care delivered, all while accelerating nursing education, competencies, and skills development [7].

According to Sun et al., a regional agenda should prioritize health system resources (mainly related to culturally relevant patient care, the nursing shortage, and standardization of training and education) [8]. Administrators setting research agendas should align with those of frontline clinicians that represent gaps in the literature. There is a need to identify the current status of National Nursing Research Priorities (NNRPs) in EMR countries and take the necessary action to cultivate these priorities.

This article was previously posted to the Research Square preprint server on April 10, 2023.

## Materials and methods

Initially, we gathered quantitative data through an online survey involving distinguished nursing and midwifery experts from Egypt, Iran, Iraq, Jordan, Pakistan, Palestine, Qatar, Oman, and Saudi Arabia. This phase laid the groundwork, offering an empirical overview of the varied facets of NNRPs across these nations.

We invited these experts directly through our regional network to share their narratives and insights to provide a richer context to our findings. This qualitative approach allowed us to delve deeper, capturing each nation's intricacies and unique challenges regarding NNRPs. These narrative submissions elucidated, expanded, or provided nuanced perspectives to our initial quantitative data.

**Figure 1:**
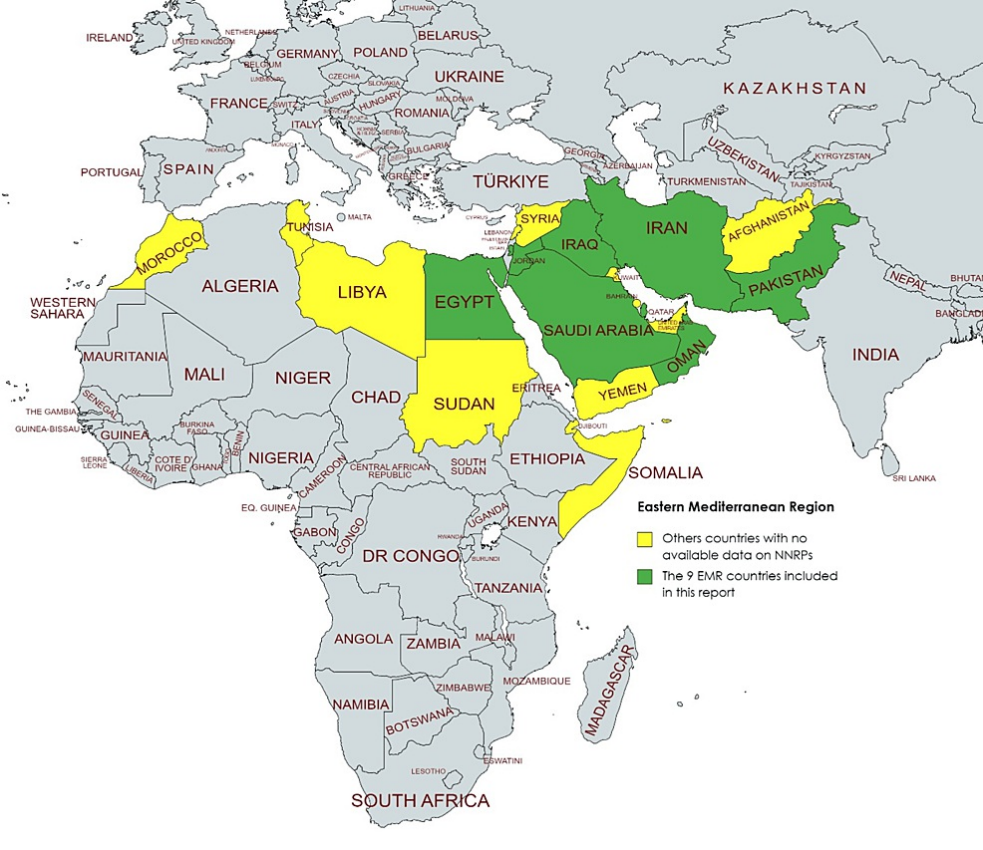
Status of the National Nursing Research Priorities in the Eastern Mediterranean Region. This figure was created by the author (Abdulqadir J. Nashwan) based on the responses received from the survey, supplemented with the authors' knowledge gained from a comprehensive review of existing literature.

By interlacing the objective metrics with subjective expert accounts, we aim to present a thorough, insightful representation of the NNRP landscape in the EMR. We believe that such a blend of data and narrative, quantitative and qualitative, offers readers a more rounded comprehension of the intricacies of our topic (Figure 1).

Seven questions are included to meet the purposes of this report (Table 1). The contributing writers' responses were analyzed, and their responses were shown graphically by frequency and percentage.

**Table 1:** Key research questions for understanding NNRPs. NNRPs, National Nursing Research Priorities.

No.	Question
1	Does your country have existing NNRPs?
2	Are your country's NNRPs published online?
3	What sources were consulted in the development of your country's NNRPs?
4	How often does your country update or revise its NNRPs?
5	Is midwifery included within the NNRPs?
6	What methodology was employed in the development of your country's NNRPs?
7	Are there any associated strategies, outcome measures/indicators, and/or funding opportunities?

## Results

**Figure 2:**
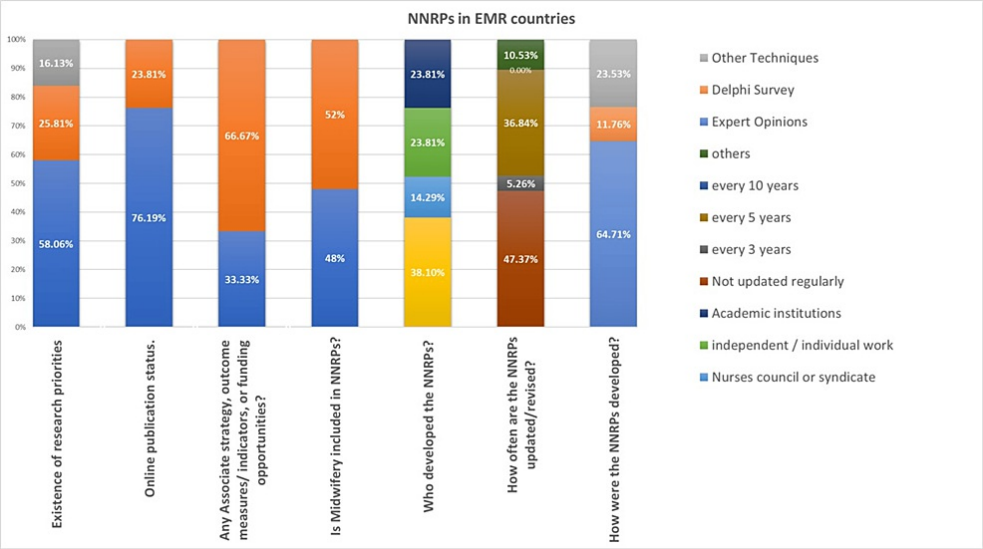
Status of the national nursing research priorities in the Eastern Mediterranean Region in numbers. NNRPs, National Nursing Research Priorities; EMR, Eastern Mediterranean Region.

A total of 31 responses were received from participants in nine EMR countries. The cumulative analysis indicates that 58% of the participating countries have existing NNRPs, while 25.8% do not, and 16% are under development. The majority (76%) were published online, and different organizations participated in developing research priorities; the higher portion (38%) was developed by the Ministry of Health (MoH) and governmental organizations, while nurses' councils and syndicates were less than 14%. Although the published research priorities need to be updated regularly, the frequency of updates is greater than every five years. Midwives were not specified in half of the published priorities as they were generally about nursing. Approximately 65% were developed by experts’ opinions and consensus, and 33% only have an associated strategy, outcome measures, and funding opportunities (Figure 2).

## Discussion

The importance of nursing research in enhancing global healthcare has been acknowledged by the WHO [7]. Identifying research priorities is crucial in countries with limited resources or healthcare emergencies, as it helps prevent redundancy and establish evidence-based practices that can improve health outcomes for the population [4,8]. The following section presents a brief review of the current status of research priorities or development for nursing and midwifery in EMR countries included in this report.

Egypt

Nursing in Egypt has changed over the last few decades. Yet, Egypt has a chronic nursing shortage [9,10]. The overall number of nurses in Egypt is believed to be 207,000, with just 56,000 belonging to the syndicate and receiving its benefits [10]. The vast majority of people are ostracized and unable to obtain fundamental rights. According to the MoH, Egypt has a shortage of 44,000 nurses at all levels [11]. This shortage affects nursing employees’ work by limiting health workforce availability and performance, and it necessitates an inventive solution by delivering the greatest benefit plans and the highest pay [12]. Half of the health workforce is composed of nurses and midwives. Numerous studies have demonstrated the critical importance of well-educated nurses in addressing the growth in infectious and chronic non-communicable illnesses and their significant contribution to improving maternal, infant, and child health [13].

Development has accelerated after the recent government recognition of nurses' contributions to healthcare access, quality, and delivery. The Egyptian government prioritizes improved healthcare services and workforce capacity. Representatives from the Technical Institutes Directorate of the Egyptian MoH and Population and the Educational Development Fund (EDF) of the Egyptian Cabinet of Ministers have acknowledged the vital importance of nurses in Egypt and emphasized the need to enhance the capacity of the nursing workforce by improving the nursing education system. They have proposed a new national curriculum as the initial step toward workforce reform and capacity building. Nevertheless, a research priority plan must be established and finalized to ensure clear direction [14].

Academic nursing research leads to the current research status in nursing. The presidency of the Nursing Sector Committee of the Supreme Council of Egyptian Universities, which supervises the promotions of nursing faculty members, conducts its research according to the research plan emanating from each college affiliated with it, which in turn emanates from the research plan of its university. Midwifery and nursing in Egypt are considered one category. They are viewed from a single perspective, where direct government supervision is located through the Central Nursing Department of the Egyptian MoH and under the supervision of the General Nursing Union [15].

Iran

Iran is a country in western Asia. It is the fourth-largest country in Asia and the second-largest country in Western Asia. Iran has a population of 86 million, making it the world's 17th most populous country and the Middle East's second largest [16].

One of the problems facing the nursing group in Iran is the need for more nurses. In Iran, there is a ratio of 2.2 nurses for every 1,000 citizens, which is nearly 5 times lower compared to the countries belonging to the Organization for Economic Cooperation and Development (OECD), with 10.4 nurses and midwives for every 1,000 citizens [17]. The deficit of nurses in Iran is anticipated to deteriorate in the next five years since many nurses are predicted to retire during this period. Furthermore, there is a projected rise in patient numbers due to Iranians' aging and population expansion [17]. The scarcity of workers and a considerable staff turnover pose difficulties in nursing and health care. To deal with the nursing shortage, some nurses were compelled to work almost 150 hours of overtime each month. The nurses expressed discontent and frustration with this excessive workload [18].

The educational system for nursing has transformed from a hospital-based program to a university-supervised system. Academic education in nursing is divided into three categories: bachelor's, master's, and doctorate. Clinical nursing is further classified into four categories: managerial, general, specialized, and primary. Every year, between 11,000 and 15,000 individuals graduate from nursing programs in Iran, while an additional 50,000 nursing graduates and undergraduate students are under the guidance of 2,000 faculty members in Iranian universities [19]. In 2013, the MoH initiated the Deputy of Nursing to improve the quality and quantity of nursing services [19]. There are approximately 33,208 midwives in Iran in various roles, including policymaking management and education (teaching midwifery students at undergraduate, master's, and doctorate levels) in various programs. A total of 10,517 midwives are working in community health centers, offering specialized care within the national reproductive health programs in collaboration with other interdisciplinary healthcare professionals. More than 20,000 midwives are working in medical facilities to aid in uncomplicated childbirths and deliver urgent midwifery services. Despite this, Iran is experiencing a shortage of midwives since the WHO has recommended a minimum of 18.1 midwives per 100,000 people [20].

The existing NNRPs encompass several critical areas to enhance the quality and effectiveness of nursing care. First, there is a strong emphasis on preparing and standardizing nursing care guidelines in patient care, ensuring consistency and high standards across various healthcare settings. Additionally, examining and presenting strategies for improving nursing education is a key priority, focusing on developing robust educational frameworks to equip future nurses better. Professional commitment and its challenges in nursing also stand out as a significant area of interest, addressing issues related to dedication and retention within the profession.

Palliative care and its challenges form another crucial priority, highlighting the need for better end-of-life care practices. Similarly, the preparation and standardization of tools for measuring the quality of care are essential to ensure accurate and effective assessment of nursing services. Research into common syndromes in patients hospitalized in intensive care units aims to improve patient outcomes and care strategies in these critical settings. Finally, investigating the quality of life of patients with chronic disorders is vital, focusing on enhancing the overall well-being and management of long-term conditions. These priorities collectively aim to advance nursing practice, education, and patient care on a national scale.

This research priority was developed by the Universities of Medical Sciences and the MoH and Medical Education through the National Iranian Nursing Research Network and published online. However, these priorities only covered nursing and did not include midwifery, which has been revised and updated.

In 2015, the NNRPs were created through a three-round Delphi method that involved 19 nursing experts in clinical practice, administration, research, and education. The priorities were subsequently reviewed and revised in 2017. The initial step involved developing a draft of nursing research issues based on input from the experts’ group and relevant evidence. Then, the list of priorities was sent to the participants in three successive phases, and directed content analysis was utilized to analyze their feedback. Later, 43 nursing experts from different regions nationwide further revised the list of priorities.

Iraq

In the past two decades, Iraq has been impacted by major armed conflicts, sanctions, and terrorist attacks. Consequently, the country is facing enormous public health challenges. The political and financial instability hindered Iraq's reconstruction of its healthcare system. The lack of healthcare services resulted in poor quality healthcare. Inaccessible data and lax security compromised the quality of Iraq's healthcare services [21].

Iraqi nurses and midwives provide essential healthcare services across all healthcare system levels, making up most of the healthcare workforce. However, nursing in Iraq has been hampered by several challenges, including multiple levels of academic preparation, negative public attitudes about nursing shared by nurses, low remuneration, and unfavorable working conditions for decades [22,23]. The institutional setting where nurses conduct their jobs may harm nurse satisfaction and patient health outcomes. Staff nurse insufficiency imposes a heavy nursing burden, which substantially affects patient satisfaction [24]. According to unpublished statistics from all of Iraq's governorates, the nurse-to-population ratio has reached an average of 22.3 nurses per 10,000 inhabitants.

Until recently, nurses had limited decision-making power in the MoH. However, there is currently a revived understanding of nursing's value. National leaders, non-governmental organizations, and United Nations organizations increasingly encourage enhancements to nurses' education, training, and professional status [25].

A robust agenda for nursing research is required to enhance health services and systems, particularly one that considers topics related to evidence-based practice. In recent years, evaluating the performance of nurses has been one of the most significant studies conducted by Iraqi nurse researchers. The literature has emphasized the quality of care and productivity [26]. Research priorities are reported annually by the Iraqi MoH and include, but are not limited to, hemoglobinopathies, quality of care, patients' satisfaction, oncology, communicable and non-communicable diseases, nosocomial infections, and mental health. Nonetheless, nursing practice and education accounted for a disproportionate number of nurses' research activities.

While Iraqi universities offer postgraduate nursing programs at the master’s and doctorate levels, an advanced practice role is not yet well established in Iraq. The biggest challenge confronting advanced nursing practice in Iraq is the lack of regulations and policies that allow nurses to practice to the full extent of their education and training, which can aid in promoting optimal job fulfillment, assessing the impact of the advanced nursing practice nursing role. Addressing barriers that impose restrictions on practice is essential to overcome those barriers and provide postgraduate nurses with licensure, privileges, and authority.

The Iraqi Nursing Syndicate (INS) was founded by Law 8/2020 and published in the official gazette on November 2, 2020. It is the entity that represents Iraqi male and female nurses, with a headquarters in Baghdad and affiliates across the governorates and Kurdistan region. The syndicate provides licenses issued to regulate nursing activities in outpatient nursing clinics. The election of the syndicate’s representatives in other Iraqi governorates is still in progress. To improve healthcare practice in the best interest of patients, the Iraqi Nursing Syndicate should endeavor to find common ground on topics such as expanding the scope of practice, boosting interprofessional collaboration, and resolving other issues.

The years of isolation and unrest in Iraq have resulted in a decline in the country's research capacity, which appears to be rebounding. At this critical juncture in the rehabilitation of the Iraqi healthcare system, there is a lack of evidence to support key decisions. Universities can develop vital cooperation with the MoH to pursue vital topics. Identifying areas of poor quality in health services and proposing solutions to rectify these shortcomings could be a first step. Data are required to better comprehend health disparities and vulnerability [27].

Given these considerations, it becomes evident that nurses have the potential to make significant advancements not only in their profession but also in fostering community development. Their unique skills and characteristics, which include the critical duty of advocacy, not only suit but also empower them to shape public policy effectively.

Jordan

Jordan is a middle-income country with a population of 11,258,30, distributed among 12 governorates over three regions (North, Middle, and South) [28]. Life expectancy in Jordan has reached 73.5 years (72.8 for males and 74.2 for females), the infant mortality rate has decreased from 23 in 2009 to 13.9 in 2012 (per 1000 live births), and the crude birth rate and the crude death rate per 1000 population in 2017 were 23.3 and 6.0, respectively. Jordan has achieved a commendable health status in the Middle East, as evident from its core health indicators. The country is regarded as a regional hub for healthcare services due to the steady rise in public and private hospitals, which currently stand at 110 with 13,731 beds. The existing hospitals and medical centers have been upgraded, and the health insurance coverage has been expanded. Treatment, medical equipment, and services have also been modernized [29]. Jordan's healthcare sector is well known for providing high-quality services on a regional and international scale, owing to the presence of world-class, internationally qualified medical personnel and accredited hospitals and facilities equipped with state-of-the-art medical machinery and equipment. In Jordan, nurses and midwives constitute 45% of the health workforce, protect the public, and ensure access to high-quality and continued health care [30]. There are 26,657 registered nurses in total, which corresponds to a ratio of 26.5 nurses per 10,000 population. Among these nurses, 58% work in the private sector, while 42% work in the public sector. Additionally, there are 3,568 midwives, corresponding to a 3.5 per 10,000 population ratio. More than half of the midwives, 52%, work in the private sector, and 48% are in the public sector [30].

Nursing and midwifery education, regulation, and practice in Jordan are provided by the Jordanian Nursing Council (JNC), which was established in 2002. One of the main objectives of the JNC is to support scientific research to enhance the profession's development. To achieve this goal, the JNC created a nursing and midwifery database in 2005 to support research development and produce high-quality research with national and international impact to improve all Jordanians' health and social well-being. The JNC collaborated with nursing schools and relevant stakeholders to identify national research priorities in Jordan. These priorities are consistent with and based on the national healthcare agenda of Jordan and the available national and international literature related to research priorities [31]. Consequently, a national strategy committee was formed, and the first national nursing strategy in Jordan (2006-2010) was accomplished. The strategy comprises eight essential dimensions, one of which is research and development, aimed at fostering a scientific research culture among nurses working in different healthcare institutions. In 2015, the JNC developed the National Nursing and Midwifery Research Priorities (2016-2020) to generate evidence that improves nursing practice and informs policymakers. The priorities also offer top-tier national strategic guidance to nurses and midwives professionals to concentrate their research interests, efforts, and investments on areas that can significantly impact public health outcomes. The NRPs for nurses and midwives in Jordan were created after consulting with researchers, leaders, and government representatives to tackle the country's health and social issues. In developing these priorities, the Joint Nursing Committee (JNC) considered the WHO's regional strategic directions for strengthening nursing and midwifery (2015-2025), as well as the WHO global health workforce (2013-2016), and WHO strategy on research for health (2013-2015). Four phases of expert discussion were conducted to determine the research priorities for nursing and midwifery in Jordan. The experts involved in these discussions represented stakeholders and policymakers in various health organizations and institutions nationwide, as well as nursing/midwifery leaders, researchers, and educators from hospitals, universities, and primary and community healthcare centers. The first phase involved a systematic review of the literature to identify the national, regional, and global health contexts related to nursing/midwifery research. The second phase involved a roundtable dialogue including 31 experts to agree on the key themes of nursing/midwifery research priorities.

The third phase involved two rounds of discussions verifying and validating the main identified research themes. During the fourth phase, research themes were ranked through group meetings and several rounds of feedback. The emerging research priorities were grouped into five primary domains: regulatory, workforce, education, leadership, and clinical practice, each with five to seven related themes. These domains align with the WHO regional domains aimed at strengthening nursing and midwifery from 2015 to 2025. Evaluation and updates of the nursing and midwifery research priorities were set to be done every two years to ensure that the issues being tackled are still the most urgent for the country and to allow for new initiatives. The strategy does not involve concrete measures, indicators, or funding opportunities. It is disseminated to all health and education institutions and can be accessed through the official JNC link (www. JNC.gov.jo). 

Pakistan

In Pakistan, there are no existing NNRPs. Pakistan has a significant shortage of nurses; only about 5% of nurses have an education level of BSc or above. In a recent national workshop on health research priority needs commissioned by WHO, nursing research was not selected as one of the top 10 priorities, mainly because the bigger priority is to tackle the capacity building of the nurses and to produce more nurses to meet the health needs of a significant population. Moreover, another issue is the physician-dominant landscape, in which nurses are underfunded and underrepresented in national health governance. Hence, there needs to be more focus on nursing research, at least in the public sector. However, to grow professionally, nurse-led research is critical [32].

Although there has been noticeable progress in the nursing profession in Pakistan in recent years, there is considerable scope for further enhancement. Despite the recognized significance of nursing within the healthcare framework, nursing research in Pakistan remains in its nascent phase. Increased funding and resources are urgently required to solidify its foundation and propel this essential field forward.

One of the main challenges facing nursing research in Pakistan is the need for more collaboration between government agencies, healthcare organizations, and universities. This has resulted in a fragmented and disconnected research landscape, with little coordination or sharing of resources. Furthermore, more trained and qualified nursing researchers are required, as is increased funding for nursing research projects.

Despite facing difficulties, there have been some encouraging advancements in the past few years. The administration has implemented measures to boost funding for research in nursing, and several universities have started providing postgraduate programs in nursing. This is helping to produce a new generation of trained nursing researchers who can contribute to the development of the field [33].

Greater collaboration between government agencies, healthcare organizations, and universities is needed to further advance the state of nursing research in Pakistan. This will require increased funding and support for nursing research and the development of a comprehensive research agenda that considers the nursing profession's needs and priorities. With these steps in place, nursing research in Pakistan can make a big difference in how well health care works there.

Palestine

Palestine is a Middle Eastern country under current Israeli occupation, with a total population of 5,290,925 million [34]. With regard to the healthcare system, there are five health service providers: the private sector, the public sector (under the MoH), non-governmental organizations (NGOs), military health services, and the United Nations Relief and Works Agency for Palestine Refugees (UNRWA). According to the National Human Resources for Health Observatory report [35] on the Palestinian health workforce, there are 39,463 practicing health workers. Furthermore, according to a WHO report [36], the ratio of nurses and midwives in Palestine was 25.7 per 10,000 population, which was lower than the target of 39 set in the country's Sustainable Development Goals. The employer for most nurses and midwives is the MoH [37].

Concerning the NNRPs in Palestine, there is no published data on this matter in Palestine. Nevertheless, the annual health report of the MoH [34] indicated that the leading causes of death among the population were cardiovascular diseases, cancer, and COVID-19, which suggested the need for more research on these illnesses. Moreover, this report highlighted the shortage in health services that were offered to people with psychological disorders; for example, it indicates that 3,607 new patients registered at different mental health centers in Palestine with an incidence rate of 73 per 100,000 population, with only 22 specialized psychiatric and community health centers providing mental health services to all the patients. This indicates the need to focus on these disorders, which is supported by the literature [38,39]. Finally, the WHO has recommended more effective antenatal care services in low- and middle-income countries, including Palestine, to reduce neonatal mortality and complications during pregnancy [40]. This suggests the need to investigate the current antenatal healthcare services and detect the outcomes of these services. 

Qatar

Qatar is a peninsula that spans approximately 22,000 square kilometers in the Gulf region. It protrudes halfway along the western shore of the Gulf, roughly 30 kilometers south of the Bahrain Islands. Over the last few decades, considerable progress has been made in Qatar's healthcare [41].

The nursing workforce in Qatar has grown significantly in recent years, driven by the country's focus on improving healthcare and the growing demand for healthcare services. The nursing workforce comprises highly qualified and experienced expatriate nurses with advanced training and education in various specialties.

Despite this growth, Qatar's nursing workforce still faces challenges. More Qatari nurses are needed [42], and more investment in nursing education and training programs is also needed to ensure its continued growth and development. To address these challenges, the Qatari government has implemented several initiatives to support the nursing workforce, including increased investment in nursing education and training programs and developing policies and programs to support and retain nursing staff. These efforts have helped improve the nursing workforce's quality of care and ensure that patients receive the care they need when needed. As a result, nursing research in Qatar has dramatically developed and improved between 2000 and 2015 [43]. In addition, a dramatic increase in publications has been noticed in the last five years, especially during COVID-19. However, despite this noticeable increase, research priorities still needed to be identified or published; even in the national health strategy, all the highlighted health priorities were medical.

The status of NNRPs in Qatar has seen significant improvement in recent years, with increased investment and focus on advancing the field. The Qatar National Vision 2030 strongly emphasizes healthcare and human development, which has helped to drive the growth of nursing research in the country [44].

One of the key areas of nursing research in Qatar is patient care, particularly improving outcomes for patients with chronic conditions. There is also a growing interest in exploring the role of technology in nursing, including the use of electronic health records, telehealth, and other digital tools.

Qatar has made significant infrastructure investments in nursing education and research, with several universities offering graduate programs in nursing and several research centers dedicated to the field [45]. Several professional organizations, such as the Qatar Nursing Association, promote and support nursing research.

However, despite these positive developments, several challenges still face nursing research in Qatar [46]. There is a need for more qualified and experienced nursing researchers, and limited funding for nursing research projects has limited researchers' ability to conduct high-quality studies and disseminate their findings effectively.

Increased research infrastructure and support for nursing researchers are needed to further advance nursing research in Qatar. This will necessitate a collaborative effort by the government, healthcare organizations, and universities to prioritize nursing research and provide the resources and support needed to drive the field forward. With these measures in place, nursing research in Qatar can significantly improve healthcare outcomes in the country.

Saudi Arabia

Saudi Arabia is part of the WHO's EMR [47]. Vision 2030 has brought Saudi Arabia a new era of advancement and wealth in health delivery, nursing, trade, education, communications, science, and technology. Bachelor's programs in nursing at KSA follow two paths: the regular nursing program (RNP) and the bridge nursing program (BNP). RNP has four years of academic education and one year of internship. BNP is a two-year curriculum followed by a six-month internship for RNs with diplomas. The Ministry of Education (MoE) oversees bachelor's programs through the 2004-founded National Commission for Academic Accreditation and Assessment (NCAAA) [48]. Midwives and nurses in Saudi Arabia have various degrees of education and training but are typically categorized within the same profession. They make vital contributions as frontline healthcare practitioners well-positioned to assess clinical research requirements [7,49].

The Saudi government faces several challenges in achieving "The Gold Standard" in nursing practice. Saudi Vision 2030 calls for nursing leaders, educators, and practitioners to work together for nursing transformation [7]. A clear plan for national research priorities is one of these transformations. Nursing research has increasingly become a measure of nursing and hospital quality, focusing on nurse participation. In addition, Magnet recognition for nursing excellence in hospitals necessitates continual internal research and practice adjustments based on nurse-generated evidence [8]. Nursing research is needed in Saudi Arabia to map the dramatic changes confronting the health service and the profession, as well as to evaluate current nursing practice and address the desire among health decision-makers to implement policies based on evidence-based studies [50,51].

Setting clear and focused priorities is a critical initial step in national research development [52]. It is becoming increasingly apparent that clinical nursing personnel are indispensable to this process. Nurses and midwives must conduct research that is relevant and up-to-date. Determining priorities is an important first step to researching the right topics and avoiding redundancy [52]. Nevertheless, there must be clear evidence of clinical nurses’ and midwives’ participation in creating a regional research agenda for the EMR [4]. However, only some national initiatives to identify research priorities have been undertaken at the level of Saudi institutions and nursing colleges.

The Saudi government offers international nursing scholarships to Saudi citizens as part of national initiatives to improve nursing education and research. About 813 Saudi students are enrolled in nursing schools outside Saudi Arabia, mostly in the US and Australia [53]. These international colleges offer nursing programs at multiple levels [48]. Also, the Saudi Commission for Health Specialties (SCFHS) has taken the lead in providing professional nursing training and preparation programs. The SCFHS website's e-learning platform offers universal training modules such as patient safety and infection control.

Recently, Alotaibi et al. (2021), in their study of setting the health research priority agenda for the MoH, referred to Health System Research Priority Themes, Service Delivery, Workforce, Information Systems, Access to Essential Medicines, Financing, Governance and Leadership, and Disaster Response [53]. Likewise, Sun et al. (2019) conducted a mixed-methods study to investigate the research priorities of clinical nurses and midwives in the EMR. Saudi Arabia was one of the investigated countries [8]. Across countries, Sun et al. (2019) identified research priorities related to the nursing workforce, health systems research, and quality of care as all critical issues that must be investigated in order to establish a solid evidence base for nursing practice [51].

Moreover, many research units at nursing colleges are now focusing on setting research priorities based on a variety of sources of information, such as reviewing the research priorities of health-related institutions and organizations, such as the WHO's Saudi Chapter (the research priorities include the Millennium Development Goals and the MoH Research and Development). The research priorities are 1) healthcare systems, 2) public health, education, and social risks, 3) diseases: burden, epidemiology, risks, prevention, and management, and 4) nursing education and practice.

The academic nursing leaders in Saudi Arabia must participate effectively in developing nursing strategies and operational plans to foster national nursing education and research development to improve the current situation. Furthermore, it is critical for politicians and planners to actively support the development of nursing as a mature and self-sufficient profession [48]. Developing local nursing research strategies and promoting local and international collaboration to conduct and use research based on nursing priorities is also necessary. More interventional nursing research is required in Saudi clinical care settings [8].

Sultanate of Oman

Oman is a Middle Eastern country with approximately 5 million people. The healthcare system in Oman is one of the best in the region, with a strong emphasis on preventive care and a well-developed network of hospitals and clinics. The government is committed to improving access to healthcare services for all citizens and provides free or subsidized services for low-income families. Private health insurance is also available for those who can afford it [54]. The MOH is keen to support the development of the nursing workforce, considered a key resource in any healthcare system. Nurses constitute more than 40% of the healthcare workers and around 60% of the health professional population [55].

The MOH has created five-year plans that provide methods and strategies to enhance the nursing workforce and has supported the development of the nursing profession since 1970. More than 40% of all healthcare employees in the sultanate and over 60% of all health professionals are nurses and midwives [55]. There is a continuous increase in the number of nurses. Since 1980, the nursing population has been growing at a rate of 4.5% annually. In 2016, 49% of the total nurse workforce, 19,760, were Omani. Fourteen thousand five hundred eighty-eight nurses work at the MoH healthcare facilities, and 65% are Omani. From 5.6 nurses per 10,000 people in 1975 to 44.8 nurses per 10,000 in 2016, the nurse-to-population ratio has grown; the Directorate General of Nursing Affairs (DGNA) is committed to providing high-quality professionals equipped with skills, knowledge, and confidence so that they can perform their roles and respond positively to change [55,56].

Nurses and midwives require a baccalaureate for entry into practice. Substantial numbers of nurses are educated to the master's level in the advanced practice role, undertaking the clinical management of complete patient care episodes with significant practice autonomy [57]. Flourishing professional associations linked to regional and international nursing and midwifery networks and professional bodies promote professional growth and status. They also contribute a professional perspective to the formulation of health policy and provide a clear professional voice in advocating for nurses, midwives, and their clients.

The official body and focal point within the MOH responsible for implementing the MoH's policy for fostering research culture in the health sector in Oman is the Centre of Studies and Research (CSR). It has been offering decision-makers, other academics, and academicians evidence-based information. Additionally, the Five-Year Health Development Plans have always stressed the importance of health research for planning and monitoring achievements [57]. Currently, there are no existing national priorities for nursing research. Nevertheless, there are common areas of research priorities for diseases and risk factors. The priority for nursing research is to promote nursing science excellence (service and education). The DGNA recognizes the significance of evidence-based practice and research. As a result, it invests in developing nursing researchers, honing their research skills, and applying those skills to address emerging issues of importance to the profession and its customers [58].

The DGNA's priorities include several strategic initiatives, and tangible steps have been taken to ensure their application. First, the development of a capacity-building network among Omani nurses interested in research is a key focus aimed at supporting quality practice. Additionally, there is an emphasis on increasing public awareness of the importance of evidence-based research and the application of research findings in clinical practice.

A significant priority is the creation of a research data bank system, which encompasses Oman-based research projects and establishes connections to local, regional, and international research facilities and literature. Regional and national researchers are supported, and various research projects are carried out in alignment with these priorities.

In accordance with "Health Vision 2050," clinical practice based on evidence and research is deemed critical for the future credibility of the nursing profession in the eyes of healthcare colleagues and the public. Achieving a high level of visibility for nursing research is essential, accomplished through publications, presentations of innovative research, and representation at the MoH.

Advanced and intensive activities focusing on evidence-based practices and research have been undertaken to empower research nurse mentors to lead and conduct research projects that address identified service and education needs. International research experts and publishers have been invited to participate in "Train the Trainer" courses as part of this effort. Fourteen nurses were trained as "Research Mentors," their roles and responsibilities included raising awareness of the importance of evidence-based research, applying research findings in clinical practice, and supervising nursing research projects within their institutions.

Limitations

Certain limitations must be considered when conducting a research paper on nursing priorities. First, data availability is a major concern. In some countries, data may not be readily available or accessible due to issues such as lack of funding, political instability, or cultural restrictions. This can lead to a lack of comprehensive information and make it difficult to draw accurate conclusions about the situation in those countries. Language barriers may also limit data availability, as translated data may not accurately reflect the original information. Furthermore, cultural differences can impact the validity of research findings, as some practices and beliefs may vary greatly between countries. These limitations must be carefully considered when drawing conclusions and making recommendations from nursing research.

## Conclusions

In summary, regardless of having a national research priority, all countries involved in this report emphasize the importance of developing nursing education and research as priorities for improving their current nursing task forces. EMR countries are challenged by the shortage of nurses, which might hinder nurses' involvement in research projects or their continued education. However, some countries, such as Jordan and Saudi Arabia, are taking a prospective approach and advocating for the future of a national research program. Most of the identified themes and concerns for the included countries are focused on medical perspectives for research priorities such as healthcare systems, public health, education, and social risks; diseases: burden, epidemiology, risks, prevention, and management; and nursing education and practice. Even though those countries have no specific research priority plan, most have a particular strategy integrated into their medical research areas or themes. 

The participating experts recommended that NNRPs be consistent with WHO regional domains on strengthening nursing and midwifery 2015-2025, including regulation, leadership, workforce, education, and clinical practice. We also emphasize that this research plan should cultivate research strategies to prepare and standardize nursing care guidelines and tools for measuring the quality of patient care, examine and present strategies for improving nursing education, maintain professional commitment in the nursing profession, and enhance palliative care and treatment of chronic disorders. Furthermore, we recommend regional collaboration between countries to set nursing research priorities for common and similar health problems. Through this collaboration, we provide shared funding and shared benefits for all contributing countries. Regional cooperation could be done through science diplomacy and, consequently, the third sustainable development goal.
